# Existential Insights in Cancer: Meaning in Life Adaptability

**DOI:** 10.3390/medicina58040461

**Published:** 2022-03-22

**Authors:** David F. Carreno, Nikolett Eisenbeck

**Affiliations:** 1 Department of Psychology, University of Almería, 04120 Almeria, Spain; carrenodf@ual.es; 2 Department of Personality, Evaluation and Psychological Treatment, University of Seville, 14004 Seville, Spain

**Keywords:** meaning in life, cancer, psychological therapy, meaning-centered psychotherapy, values

## Abstract

Previous research demonstrated that the cancer diagnosis and treatment evoke existential concerns, especially ones related to meaning in life and meaning-making processes. The cancer experience is a vital challenge that often entails changes in what is personally important in life. Firstly, this paper collects evidence supporting that meaning adaptability, the way people adapt their meaning in life to the cancer experience, is a central element in the mental health of cancer patients. Various theories that could account for this meaning adaptability are introduced. Secondly, the paper provides a compilation of studies showing how people change what is significant in their lives within the course of cancer. Finally, the paper presents the available psychological therapies directed to facilitate meaning adaptability in this population. Meaning-centered interventions have been demonstrated to be effective in alleviating the suffering related to the cancer experience and promoting personal growth.

## 1. Existential Concerns in Cancer

Cancer is a leading cause of death worldwide, showing a prevalence of 565.7 cases per 100,000 inhabitants in 2020 [1]. It is one of the most difficult challenges in the lives of millions of people, affecting all levels of human functioning, including various physiological, psychological, and social aspects. Especially, cancer inherently puts at the forefront one’s mortality, and thus, it is related to various existential fears [2]. Existential concerns among cancer patients are prevalent and have been studied in the past [3]. These concerns include, among others, meaning in life and meaning-making processes; faith, belief, and spirituality; love and belonging; forgiveness and giving; and peace and harmony [3,4,5].

For instance, religious and spiritual beliefs can become quite salient after a cancer diagnosis, as they can act as a framework for accepting and understanding the challenges and difficulties that the person experiences [6]. Spirituality as a broader concept than religion encompasses “the way in which people understand their lives in view of their ultimate meaning and value” ([7], p. 336). Salutary outcomes of spirituality in individuals with cancer are generally documented in the literature, showing negative associations between spiritual well-being and depression, hopelessness, and end-of-life despair, among others (e.g., [8,9,10]). Measurement of spiritual well-being has become an important tool for the assessment of the quality of life among cancer patients [11]. According to a previous study, it is divided into two main components, namely meaning/peace and faith [11]. Faith can be understood as the sensation of comfort derived from being connected to something bigger than the self, while the meaning/peace component is related to a sense of meaning, peace, and purpose in life [11]. In a longitudinal study [12], faith was associated with increased cancer-related growth, while the meaning/peace component was related to fewer depressive symptoms and higher vitality among people with breast cancer across 12 months. Meaning/peace was also associated with improved mental health and less cancer-related distress.

Overall, the aforementioned studies show that meaning in life and meaning-making processes seem to be one of the most relevant existential concerns during the cancer experience [3]. This article summarizes research on this topic and proposes meaning adaptability as a separate construct to be studied because of its clinical potential in the oncological population.

## 2. Meaning in Life in Cancer

One of the most accepted definitions of meaning in life understands it as the “sense made of, and significance felt regarding, the nature of one’s being and existence” ([13], p. 81). There is a certain scholarly agreement that the three primary components of meaning in life are coherence/comprehension, purpose, and significance/existential mattering [14,15]. That is, “lives may be experienced as meaningful when they are felt to have significance beyond the trivial or momentary, to have a purpose, or to have a coherence that transcends chaos.” ([16], p. 180). Besides these three components, another crucial aspect of meaning is responsibility: the commitment to actions related to personal values (i.e., actions that the person thinks are morally right) [17]. In this sense, meaning in life is not only about the cognitions and emotions regarding one’s purpose and significance in life but also about whether one behaves according to one’s personal values. Indeed, when this behavioral aspect is included in the understanding of meaning, the influence of this construct on other psychological variables seems to be higher than when only cognitive and emotional aspects are considered [18].

This multidimensional conceptualization of meaning (including coherence, purpose, significance, and responsibility) seem to be central in cancer: the diagnosis and treatment of this illness entail an existential plight, as the person’s whole belief system, the purpose of life, identity, and values-based actions are called into question [19]. Such an existential crisis triggers a search for meaning in most cases [19]. According to Park et al. [20], these meaning-making efforts may lead patients towards better adjustment when they achieve the creation of adaptive meanings as a result of the cancer experience (the sense of growth, increased life meaningfulness, and restored just-world belief). In this line, there are two ways of making meaning in the context of cancer. The first one is through making situational meaning: people can assimilate the cancer situation within their existing meaning in life [21]. This type of meaning-making is more related to comprehensibility, that is, the cognitive assimilation of new trauma-related information into a pre-existing worldview. The second way of meaning-making happens through the adaptation of global meaning in life to the cancer conditions: people can accommodate or change their worldview to integrate the cancer experience. This second via of meaning-making is more associated with the “significance” aspect of meaning (the question about what matters in life). The present paper is focused on this second type of meaning-making.

Paradoxically enough, mortality salience related to cancer (the acknowledgment of personal death) has the potential to make us not only think about meaning but possibly to lead to a more meaningful, authentic, and fulfilling life [22]. Data show that meaning in life is negatively associated with depression, anxiety, and feelings of distress, while it is positively related to psychological well-being during the cancer experience [23,24]. These observations are supported by a longitudinal study that establishes global meaning as a negative predictor of demoralization and depression [25].

On the contrary, the loss of meaning can be related to several adverse outcomes. Approximately 17% of cancer patients reported a desire to terminate their life not because of their pain levels but due to meaninglessness, loss of hope, and depression [26]. Other studies confirmed that, among people with advanced cancer, meaninglessness was closely related to a desire for hastened death [27], and it mediated the relationship between physical impairment and the wish for a hastened death [28].

These findings show that the adaptability of meaning in life is closely related to the wellbeing of cancer patients. While a significant part of the literature has investigated the role of meaning-making in the adjustment to cancer, such as the research on post-traumatic growth [29,30], there is still a reduced number of studies exploring quantitatively the vast amalgam of life areas and personal values that may change in importance following a cancer diagnosis. In the next sections, there is an updated compilation of these studies and a presentation of various theories that might explain the impact of cancer on meaning in life. Finally, those therapies aimed to facilitate meaning-making in this population are presented.

## 3. Sources of Meaning in Cancer: Shift and Reprioritization

Sources of meaning, those personal values or valued life areas from where one derives a sense of coherence, purpose, mattering, and responsibility, are inherently subjective and individual. However, in the general population, the most frequently mentioned contribution to meaning in life seems to be personal relationships [31,32,33,34], followed by a variety of other sources, such as personal growth, self-actualization, work, achievement, creativity, self-transcendence, generativity, or religion, among others [34,35,36,37,38,39].

Similar to the healthy population, relationships and contributions to the welfare of other people seem to be at the forefront of the experience of meaningfulness among cancer patients [24,40]. In addition, there are some data available showing that, as compared to people without cancer, terminally ill patients reported interpersonal relationships higher on their list of sources of meaning [23].

Other personally valued areas from which people derive meaning can also differ in importance between people with cancer and the general population. A previous study reported that the highest-rated personal values among terminally ill people were benevolence, universalism, and self-direction, while power, achievement, and stimulation were the lowest rated [41]. Cancer patients showed higher levels of benevolence and lower levels of self-enhancement values than their healthy counterparts [41].

These differences in sources of meaning compared to the general population may indicate a shift related to the personal values system and quality of life as a result of the cancer experience [42,43]. This response shift has been defined as “a change in the meaning of one’s self-evaluation of quality of life as a result of changes in internal standards, values and the conceptualization of quality of life” ([44], p. 1509). For instance, according to an earlier study [43], around half of the patients shift their priorities from one area to another due to cancer. In particular, family and health seem to become more important over time. This shift was only found to be beneficial (i.e., leading to a better adjustment to the illness) for those who shifted from an unfavorably rated life domain to a more positively rated one [43]. Response shift was used as a marker to evaluate treatment effects [44], and it is related to the quality of life of cancer patients [43,45,46]. However, there are few data on how exactly people change their priorities during the cancer experience. For instance, Greszta and Siemińska [47] showed that after the diagnosis, a heterogeneous group of cancer patients rated religious morality, personal orientation, self-construction, family security, and delayed gratification higher than before, while some values, such as immediate gratification, self-expansion, and competence, decreased in importance.

The response shift can be so substantial for certain people that it can even be labeled as post-traumatic growth (PTG) [29]. That is, apart from the negative consequences of cancer, people can experience increased personal strength, enhanced life appreciation, new possibilities, reevaluation of former priorities, closer relationships with others, and positive spiritual changes due to changes in their priorities and actions [29,30].

Although it seems like that a great number of people adapt their sources of meaning to the cancer condition in a clinically beneficial way, there are only a handful of theories that have been formulated to precisely describe and explain the changes in meaning in life and the sources of meaning during the cancer experience. These theories are presented next.

## 4. Theories on the Impact of the Cancer Experience on Meaning in Life

According to one of the most prominent theories in the area, the Terror Management Theory (TMT) [48,49], death reminders trigger death anxiety and terror, which in turn activates defense mechanisms, for instance, escapism and the reaffirmation of cultural beliefs. This theory thus hypothesizes that the key motivator for the shift in personal values among cancer patients is the management of death anxiety.

Another explanation for the impact of cancer on meaning in life is presented by the Meaning Management Theory (MMT) [50]. According to this approach, death reminders do not only trigger defense mechanisms as described by the TMT but also can evoke other processes, such as the search for meaning, life appreciation, and a quest for a more purposeful life. Defensive mechanisms serve a protective function by seeking security and self-preservation, while the search for meaning and life appreciation serves a growth-related function, as it creates opportunities for personal development. This model thus postulates the existence of a dual existential system. As indirect support for this theory, data show that confronting death with acceptance instead of defense mechanisms/avoidance seems to be related to lower levels of anxiety and existential distress [51], while denial and avoidance have been associated with psychological maladjustment and loss of meaning in life [52,53]. Explicit death thoughts can indeed trigger growth-related processes, for instance, greater spirituality, heightened focus on intrinsic goals, and inspire actions towards a more meaningful life (for a review, see [54], and for the dual-existential systems model, see [55]).

Notably, one of the core processes described by the MMT as promoting meaning is the so-called meaning-reconstruction. Meaning-reconstruction involves active meaning-seeking, meaning-making, and personal transformation to reinstate a sense of coherence and order when certain events challenge one’s worldview, core values, or life goals. This theory affirms that the adjustment to changes in life is facilitated by finding meaning in them and by discovering benefits amidst the suffering. These ideas are in line with Viktor Frankl’s work on meaning stating that meaning can be found even under the most difficult circumstances [56].

Other theories, such as the Self-Determination Theory (SDT) [57] and the Socioemotional Selectivity Theory (SST) [58], can also add information to the explanation of the existential impact of cancer. SDT postulates that the quest for self-esteem echoes the pursuit of satisfying the basic psychological needs of competence, relatedness, and autonomy. According to this theory, when facing suffering and death, personal development is more about the intrinsic motivation for growth than about the defense against anxiety [57]. On the other hand, SST argues that time perception changes the sources of motivation. That is, when time in life is seen as limited, personal fulfillment is not focused on long-term goals but rather on the regulation of present affect, such as the maximization of meaningful activities, for instance, deepening existing relationships [58,59].

Finally, the concept of trait mindfulness, defined as the disposition of attentiveness to the present moment and a nonjudgmental acceptance of the moment-to-moment experience (see [60,61,62]), may also add to the explanation of the relationship between the cancer experience and meaning in life. Higher levels of trait mindfulness seem to predict fewer defenses against death anxiety as evidenced by less death-thought suppression, lower defenses of one’s worldview, and less striving for self-esteem after a mortality awareness induction [63]. These results may indicate that mindfulness could facilitate meaning-making processes.

Although there is still a great deal that is unknown regarding how meaning in life and sources of meaning are adjusted due to cancer, there is a growing number of psychological interventions aimed at facilitating meaning adaptability and fostering global meaning in life.

## 5. Meaning-Centered Interventions in Cancer

Therapeutic interventions that aim at improving meaning, meaning-making, and spiritual well-being can potentially be beneficial for cancer patients. These therapies, implicitly or explicitly, promote a re-evaluation of life priorities (and a possible response shift) to be able to adapt to the cancer experience. These therapies include, for instance, different iterations of the meaning-centered psychotherapy (in individual or group format) [64,65,66], the Managing Cancer and Living Meaningfully intervention (CALM) [67], and the dignity therapy [68]. These therapies have been generally successful in positively impacting areas related to quality of life, spiritual well-being, meaning in life, and sense of dignity, while decreasing markers of psychopathology, such as depression, anxiety, and the wish to hasten death (e.g., [3,64,65,66,68]).

For instance, the effect of meaning-centered individual therapy was greater than the effect of treatment as usual for quality of life and sense of meaning (*d* = 0.19), [64]. Another randomized trial [69] showed that a meaning-centered group intervention among cancer patients significantly improved meaning in life (*d* = 0.81), goal focus (*d* = 1.07), positive relationships (*d* = 0.59), purpose (*d* = 0.69), and fighting spirit (*d* = 0.61) while decreasing helplessness (*d* = −0.87), distress (*d* = −0.6), and depression (*d* = −0.38) as compared to usual care. Similarly, an earlier meaning-making treatment intervention as compared to routine care enhanced optimism, self-esteem, and self-efficacy among people diagnosed with colorectal/breast cancer [19]. Dignity therapy also seems to be successful: in an intervention, 76% of the cancer patients reported heightened sense of dignity, 67% mentioned an increased sense of meaning, and 47% noted more will to live [68].

Therapies focusing on personal values and purpose can also be beneficial in the adjustment to living with cancer. The Acceptance and Commitment Therapy (ACT) [70] has an emphasis on accepting unwanted thoughts and feelings and promoting actions in accordance with personal values. ACT showed to be effective at increasing quality of life, positive emotional states, and psychological flexibility among cancer patients [71,72]. In addition, other therapies that indirectly promote meaning in life, such as mindfulness-related therapies, can be beneficial (for a review of the utility of mindfulness in cancer, see [73]).

Overall, the available research on meaning-related therapies has provided evidence on the importance of this topic in cancer. The success of these therapies indicates that focusing on sources of meaning in life can be valuable in enhancing the quality of life of this population.

## 6. Conclusions and Future Directions

The prevalence of cancer worldwide is alarming. Nearly one in six deaths were due to cancer in 2020 [1]. This statistic itself, with the myriads of economic and social consequences it entails, puts cancer as one of the most pressing issues of our society. Cancer diagnosis and treatment inevitably raise questions about the meaning of one’s existence. People tend to respond to these questions by adjusting their sources of meaning, and therapeutic interventions seem to help them to adapt to their new reality.

However, data on meaning-making processes during the cancer experience are still scarce. For instance, studies are needed to understand the neural correlates of meaning-making processes in cancer. Furthermore, it is not clear how exactly changes in meaning in life and the flexibility of the individual to modify their sources of meaning during the cancer experience relate to their psychological and physical well-being. There are also few quantitative data on the number of sources of meaning and personal values that change in importance among cancer patients as well as a lack of controlled studies demonstrating the extent to which those people who restructure their sources of meaning are or are not better off psychologically. Evidence to date suggests that meaning adaptability is a key factor in the healthy adjustment of the cancer experience, as it is a significant precursor for post-traumatic growth. However, more studies focusing on the flexibility and adaptability of meaning in life and the sources used to sustain it are needed.

## Data Availability

Not applicable.
